# Overall survival of patients with metastatic breast cancer in Sweden: a nationwide study

**DOI:** 10.1038/s41416-022-01845-z

**Published:** 2022-05-21

**Authors:** Antonis Valachis, Peter Carlqvist, Yuanjun Ma, Máté Szilcz, Jonatan Freilich, Simona Vertuani, Barbro Holm, Henrik Lindman

**Affiliations:** 1 Department of Oncology, Faculty of Medicine and Health, Örebro University Hospital, Örebro, 701 05 Sweden; 2 Nordic Market Access AB, Stockholm, 113 59 Sweden; 3 Parexel International, Stockholm, 103 59 Sweden; 4 Department of Medical Epidemiology and Biostatistics, Karolinska Institutet, Stockholm, 171 77 Sweden; 5 Department of Public Health and Clinical Medicine, Umeå University, Umeå, 901 87 Sweden; 6 Novartis Sverige AB, Kista, 164 28 Sweden; 7 Department of Immunology, Genetics and Pathology, Experimental and Clinical Oncology; Clinical Oncology, Faculty of Medicine, Uppsala University Hospital, Uppsala, 751 85 Sweden

**Keywords:** Breast cancer, Oncology

## Abstract

**Background:**

Breast cancer is the most common cancer among women in Sweden. Whereas survival for the overall breast cancer population is well-documented, survival of patients with metastatic breast cancer (MBC) is harder to quantify due to the lack of reliable data on disease recurrence in national cancer registers.

**Methods:**

This study used machine learning to classify the total MBC population in Sweden diagnosed between 2009 and 2016 using national registers, with the aim to estimate overall survival (OS).

**Results:**

The total population consisted of 13,832 patients—2528 (18.3%) had de novo MBC whereas 11,304 (81.7%) were classed as having a recurrent MBC. Median OS for patients with MBC was found to be 29.8 months 95% confidence interval (CI) [28.9, 30.6]. Hormone-receptor (HR)-positive MBC had a median OS of 37.0 months 95% CI [35.9, 38.3] compared to 9.9 months 95% CI [9.1, 11.0] for patients with HR-negative MBC.

**Conclusion:**

This study covered the entire MBC population in Sweden during the study time and may serve as a baseline for assessing the effect of new treatment strategies in MBC introduced after the study period.

## Background

Breast cancer is the most common cancer among women in Sweden—in 2017, ~8000 women were diagnosed with breast cancer and 1400 women died due to the disease [1]. Overall survival (OS) for breast cancer patients is well-reported and comparisons between countries are readily available online through the collaboration of nationwide cancer registries [2–4]. However, the study of OS for patients with metastatic disease is problematic, due to difficulties in identifying patients with distant recurrence of breast cancer in available registries [5].

Close to one-third of patients diagnosed with early breast cancer (Stage I–III) will develop metastatic breast cancer (MBC) with distant metastases [6]. The disease-free or metastasis-free intervals may range from months to several years [7]. However, once distant recurrence has occurred, the disease is considered to be incurable and median OS for metastatic patients is reported to be between 2 and 3 years [8]. In addition to patients diagnosed with early breast cancer prior to metastases, 3–5% of patients are diagnosed with metastatic disease (Stage IV, de novo MBC [dnMBC]) [9, 10]. Patients diagnosed with dnMBC and patients with distant recurrent disease (rMBC) may have different prognoses due to both differences in the biology of their breast cancer and response to treatment [11]. It is, thus, essential to be able to distinguish between these subtypes [6, 11]. The clinical course of patients diagnosed with rMBC or dnMBC is highly variable, with some patients dying within months of diagnosis and others living for 10 years or more [11]. Several prognostic and predictive factors influence the clinical course of MBC including factors reflecting the tumour biology, such as hormone-receptor (HR) status and human epidermal growth factor receptor 2 (HER2) status, but also age, performance status, and the location and number of distant metastases [12, 13].

The Swedish Cancer Register, similar to most population-based and nationwide cancer registries [14], lacks specific information on breast cancer recurrence. To be able to estimate the overall MBC population, a previously developed machine-learning algorithm was used to classify the MBC population in Sweden [15].

The objective of this retrospective registry study was to analyse the OS of the machine-learning-based classified MBC population in Sweden, thus overcoming the challenge of identifying MBC due to lack of information on recurrent disease. Considering the recent improvements in OS for patients with MBC due to new treatment options [16], the results of the present study could serve as a baseline to explore the effectiveness of new targeted treatments in a real-world setting.

## Methods

The study was a retrospective, observational study using Swedish secondary national registry data. The study was approved after vetting by the Regional Ethical Review Board in Stockholm, Sweden (2017/424). To enable the identification of data for patients with MBC (de novo and recurrent metastatic) in Swedish nationwide administrative health registries, a previously developed machine-learning algorithm or “classifier” was used. Detailed information regarding the development of the classifier is provided elsewhere [15]. In brief, a support vector machine (SVM) classifier was trained and tested utilising a local registry of breast cancer patients at the university hospital in Uppsala. The Uppsala cohort (*n* = 3899) was linked to four health registries with national coverage in Sweden: the Swedish Cancer Register, the National Patient Register, the Prescribed Drug Register, and the Cause of Death Register, using a personal identification number (PIN). The PIN is a unique number assigned to every individual living in Sweden allowing the linkage of data variables between various registries. The Swedish Cancer Register registers all new primary malignancies in Sweden and contains patient information together with information on the malignancy: site; histological type; method of diagnosis and date of diagnosis [17]. The National Patient Register collects information on hospital visits and is updated monthly. It includes information on the diagnosis codes associated with each visit or hospital stay and procedure codes, as well as socio-demographic information such as age and sex. The Prescribed Drug Register records the national dispensing of prescribed and reimbursed outpatient drugs (drugs administrated outside of a hospital or a clinic, typically oral treatments and self-administrated subcutaneous injections) and includes variables on the type of drug: Anatomical Therapeutic Chemical (ATC) code, formulation, generic and brand name, and information about pack size. The Cause of Death Register reports the date and cause of death. The quality of the Swedish national administrative registries is high and the National Board of Health and Welfare (NBHW) reports close to complete coverage [18]. The key features selected by the SVM classifier were codes for secondary neoplasm: M1, C78, C79, in the Swedish Cancer Register and the National Patient Register, respectively.

The previously developed SVM classifier was used to identify data for patients with MBC in the national registries from amongst all patients who were diagnosed with breast cancer between 2009-01-01 and 2016-12-31. The identified MBC population was subsequently analysed for OS. Patients with distant metastases at breast cancer diagnosis or who were diagnosed with metastatic disease before or within 3 months from the diagnosis of the primary breast tumour, were classified with dnMBC. Treatment with an endocrine drug was used as a proxy to identify patients with HR-positive disease. HR-positive disease was defined as the presence of at least two prescriptions of ATC codes: L02BG04 (letrozole), L02BG06 (exemestane), L02BG03 (anastrozole), L02AE03 (goserelin), L02BA01 (tamoxifen), L02BA03 (fulvestrant), L02AB01 (megestrol), G03CA03 (estradiol), L02BA02 (toremifene). A similar approach for the identification of HER2 status was not possible as the HER2-targeted treatments (e.g., trastuzumab) are administrated in the hospital and not recorded in the Prescribed Drug Register.

Overall survival (OS) was analysed using the Kaplan–Meier estimator. The difference between survival curves was estimated using the log-rank test, with a chosen significance level of 0.05. Start of follow-up was the date of MBC diagnosis and the patients were followed until death or until end of follow-up at 2016-12-31. A Cox proportional hazard (PH) model was used to analyse differences in the rate of risk accumulation, summarised by the hazard ratio (HzR), the available variables for regression was diagnosis (dnMBC vs. rMBC) age, and HR status. All analyses were performed using R (version 3.6 [19]) and SAS software, Version 9.3 for Windows. Copyright © [2014] SAS Institute Inc. SAS and all other SAS Institute Inc. product or service names are registered trademarks or trademarks of SAS Institute Inc., Cary, NC, USA.

## Results

The SVM classifier identified a total of 13,832 patients with MBC, 9.2% of all patients in Sweden diagnosed with breast cancer between 2009 and 2016. A total of 13,824 had an estimated date of MBC diagnosis and could thus be included in the survival analysis. As previously reported [15], of the total MBC population, 2528 (18%) were classified as dnMBC and 10,497 (76%) were identified as having HR-positive disease.

**Fig. 1:**
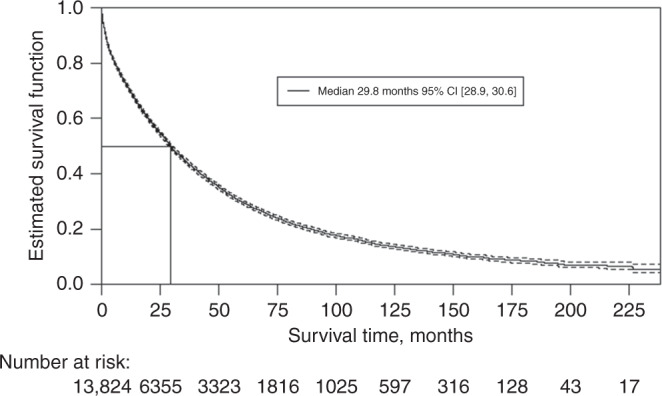
Overall survival from metastatic breast cancer diagnosis.

Building on our previous report, our current study found that the median OS for the overall MBC population was 29.8 months 95% CI [28.9, 30.6] from diagnosis of MBC (Fig. 1), and the 2-year survival rate was 56% 95% CI [55%, 57%], decreasing to 14% 95% CI [14%, 15%] at 10 years (Table 1).

**Table 1:** Landmark overall survival rates.

Landmark OS (%, 95 CI)	MBC, total (*n* = 13,826)	MBC de novo (*n* = 2528)	MBC recurrent (*n* = 11,298)	MBC HR-positive (*n* = 10,497)	MBC HR-negative (*n* = 3,329)	MBC age of diagnosis, <50 years (*n* = 1777)	MBC age of diagnosis, 50–70 years (*n* = 6082)	MBC age of diagnosis, >70 years (*n* = 5967)
2 years	56 (55–57)	56 (54–58)	56 (49–55)	63 (62–64)	32 (31–34)	68 (66–70)	62 (61–63)	46 (44–47)
3 years	45 (44–46)	46 (44–48)	45 (44–46)	51 (50–52)	25 (23–27)	55 (53–58)	51 (50–52)	35 (34–36)
5 years	30 (29–31)	31 (29–33)	30 (29–31)	34 (33–35)	19 (17–20)	40 (37–42)	35 (34–36)	22 (21–23)
10 years	14 (14–15)	16 (14–18)	14 (13–15)	15 (14–16)	12 (11–13)	22 (20–25)	18 (17–20)	7 (7–9)

*MBC* metastatic breast cancer, *HR* hormone status, *OS* overall survival, *CI* confidence interval.

**Fig. 2:**
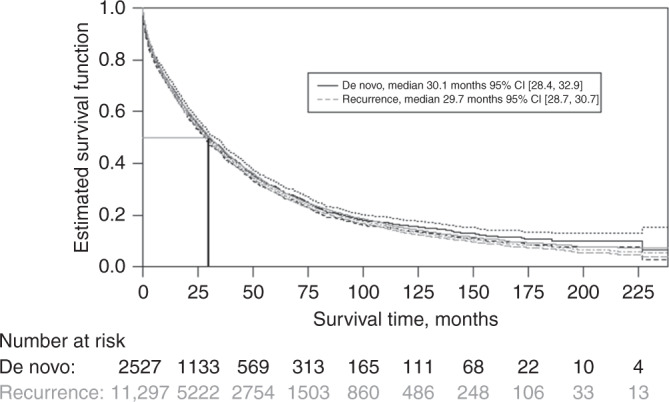
Overall survival from metastatic breast cancer diagnosis, stratified by stage at diagnosis.

The median OS was found to be 30.1 months 95% CI [28.4, 32.9] for dnMBC and 29.7 months 95% CI [28.7, 30.7] for rMBC (Fig. 2). Multivariate Cox regression analysis, adjusting for age and hormone-receptor status, estimated the HzR for OS for dnMBC versus rMBC to 0.92 95% CI [0.87, 0.97] (Table 2).

**Table 2:** Results of the Cox regression model.

Covariate	HzR	95% CI	*P* value
De novo (vs recurrence)	0.92	0.87, 0.97	*P* = 0.0016
Age (as continuous variable)	1.03	1.02, 1.03	*P* = 0.0001
HR-positive (vs HR-negative)	0.50	0.48, 0.52	*P* < 0.0001

*HR* hormone receptor, *HzR* hazard ratio, *CI* confidence interval.

**Fig. 3:**
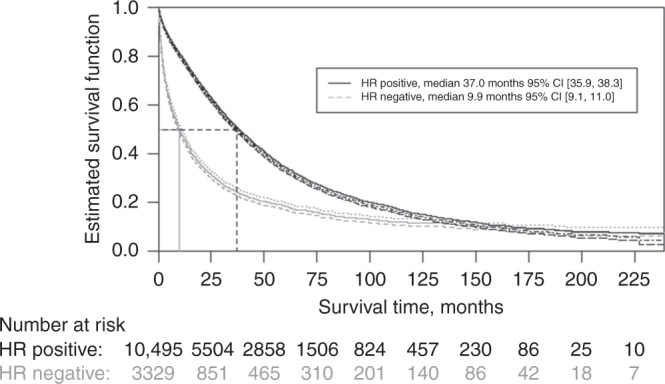
Overall survival from metastatic breast cancer diagnosis, stratified by hormone-receptor status.

The cohort with HR-positive disease had a prolonged OS compared with the HR-negative cohort, with a median OS of 37.0 months (95% CI [35.9, 38.3] and 9.9 months 95% CI [9.1, 11.0], respectively (Fig. 3). A total of 34% 95% CI [33%, 35%] of patients with HR-positive disease were alive at 5 years after diagnosis compared with 19% 95% CI [17%, 20%] of patients with HR-negative disease. In multivariate Cox regression analysis, the HzR for OS for HR-positive versus HR-negative disease was 0.50 95% CI [0.48–0.52], see Table 2.

**Fig. 4:**
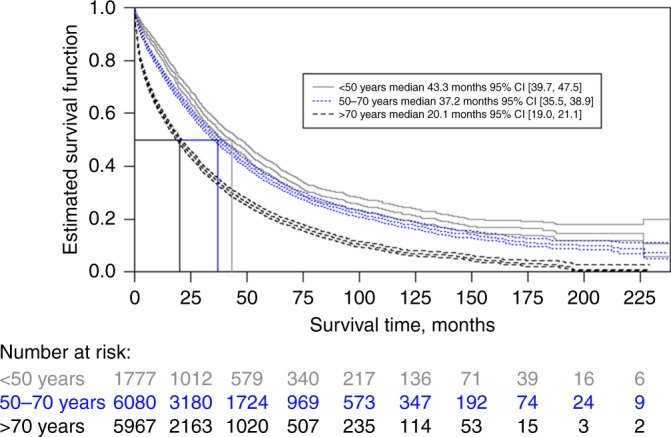
Overall survival from metastatic breast cancer diagnosis, stratified by age (age group) at diagnosis.

Median OS declined with increasing age at diagnosis. The youngest age group (less than 50 years at MBC diagnosis) had a median OS of 43.3 months 95% CI [39.7, 47.5] compared with 37.2 months 95% CI [35.5–38.9] for patients diagnosed between 50 and 70 years of age, and 20.1 months 95% CI [19.0, 21.1] for patients diagnosed after 70 years of age (Fig. 4). The 5-year OS for patients aged below 50 years at diagnosis was 40% compared with 35% for those diagnosed at ages 50 to 70 years, and 22% for patients diagnosed after the age of 70 years (Table 1). The HzR for age was found to be 1.03 95% CI [1.02, 1.03], for each additional year of age at diagnosis the hazard of death increased by 3% (Table 2).

Whereas the multivariate Cox regression found that age, dnMBC versus rMBC, and HR status had an association with the hazard of death (Table 2), the largest effect on OS was HR status, with an estimated 50% reduction in hazard (HzR = 0.50) over the study period for those who were HR-positive compared with those who were HR-negative.

## Discussion

Nationwide population-based health registries are a valuable source for the study of incidence, prevalence, and disease outcomes, offering a way to follow the clinical course of unselected patients in a real-world setting. With the use of modern machine-learning techniques and a unique PIN, it was possible to identify and link data for Swedish patients with MBC between registries to leverage the detail contained in local registries with the coverage of national registries. This study aimed to identify the entire MBC population in Sweden (i.e. both patients with distant recurrence and patients with disseminated disease at diagnosis) and assess their OS. The survival analysis showed that patients diagnosed with MBC in Sweden between 2009 and 2016 had a median OS of 29.8 months from diagnosis. The study also showed that the 5-year survival for this cohort was 30% and a small proportion of patients with MBC were found to have prolonged survival, with a 10-year OS rate of 14%.

Comparing OS rates in our study with OS rates for patients diagnosed between 2000 and 2004 in an earlier study in Sweden [20] (which covered approximately 20% of the Swedish population) where median OS ranged from 14.5 to 16.1 months and 5-year OS was 15.2%, the results of the present study show a longer median OS and a higher 5-year OS rate. Although comparing studies with different methodologies should be done with caution, an improvement in OS over time could potentially be explained by advances in the treatment strategy for MBC. In fact, a more recent Swedish study with a smaller MBC cohort from the County of Kalmar (*n* = 784), found an improvement in the median OS from 13 months to 33 months during the period 1985 to 2014, and an increased 5-year survival rate from 10 to 27% [21]. The improvement in survival of patients with MBC over time has also been reported in a recent meta-analysis [16]. The increase in survival may be attributable to the development and introduction of new anticancer agents in clinical practice most notably endocrine [22] and anti-HER2 therapies [23]. This highlights the importance of this study as a baseline to explore the survival impact of recently introduced treatments such as CDK4/6 inhibitors [24] for HR-positive MBC and the future introduction of PIK3CA-targeted [25] therapy in Sweden. Furthermore, longer survival times urge the need to re-organise the palliative care for breast cancer patients with focus on maintaining the quality of life and managing other comorbidities, which will become increasingly important as patients live longer with their metastatic disease.

The small difference in median OS between patients with de novo disease and those with recurrent disease is in line with a study by Lobbezoo et al. [6] that reported that patients with dnMBC had similar survival outcomes to a subgroup of patients with rMBC (i.e. the subgroup which had a prolonged disease-free interval of more than 24 months). In addition, Weide et al. reported no statistically significant difference between patients with primary metastases and patients whose metastases occurred after adjuvant therapy [26].

However, our findings differ from those of Malmgren et al. [11], who reported that patients with dnMBC had a better prognosis compared with those with rMBC, albeit in a smaller cohort of patients than ours (*n* [dnMBC] = 247, *n* [rMBC] = 911), and based on disease-specific survival (DSS), rather than OS. Patients with dnMBC were reported to have a 5-year DSS of 44% compared with 21% for patients with rMBC (*P* < 0.001). Furthermore, in a study by den Brok et al., [27] increased OS was found for patients with dnMBC compared with those with rMBC, irrespective of HR- and HER2 status (*n* = 3645 with known HR status, of which *n* = 2796 had known HER2 status). The hypothesis that there is a difference in survival between dnMBC and rMBC is based on that dnMBC is associated with attributes with less negative impact on the prognosis of survival. De novo disease is more likely to have characteristics such as being oligometastatic with bone metastasis only, more often HR-positive and treatment naïve (i.e. having no opportunity to develop resistance to treatment) [27].

Due to limited information regarding the distribution of molecular subtype, i.e. HER2 status on the identified MBC population in this study, it is difficult to further explore the reasons for the small differences in median survival between dnMBC and rMBC compared to other studies. One explanation could be the difference in the proportion of patients with HR-positive disease between the two groups: the proportion of patients with HR-positive disease in our study was 60.8% of those with dnMBC, and 79.3% of those with rMBC and a Cox multivariate regression analysis revealed a small survival advantage for patients with de novo disease versus those with distant recurrence (HzR = 0.92, 95% CI [0.87, 0.97]) when controlled for age and HR status. HR status is a known prognostic factor for OS as well as a predictive factor for response to endocrine therapy [6].

In line with other studies [11, 20], this study found that older age at diagnosis was associated with a worse prognosis, with a median survival of 43 months for patients diagnosed below 50 years versus 20 months in patients diagnosed above 70 years. The Cox regression analysis showed that each additional year of age at diagnosis increased hazard of death by 3%, similar to the findings of Weide et al. (1.5%) [26] and Lobbezoo et al. (2%) [6].

The current study showed that HR status had the largest impact on OS, compared with the other variables assessed (age and dnMBC/rMBC status). Patients with HR-positive disease had a median OS nearly four times that of patients with HR-negative disease (37 versus 10 months). Our finding of longer survival for patients with HR-positive disease is similar to other studies, and these survival differences are potentially attributable to both the observation that HR-positive disease is the less aggressive of the two disease types, and the availability of endocrine treatments (with or without anti-HER-2 therapy) for patients with positive HR status and HER2 disease [6, 20, 26, 28].

The study had limitations. The results should be interpreted in light of the limitations associated with the classifier used to identify the patient population, as reported previously [15]. The patient population may include false positives and patients with MBC treated outside of specialist care will not have been included. In addition to this, it was not possible to identify other factors that may affect OS, beyond the HR status of the tumours, such as comorbidities, HER2 status, site of metastasis, metastatic tumour burden, and the use of prior therapy for breast cancer, as these variables are not available in the Cancer Register. Therefore these factors were not included in the Cox regression. In particular, HER2 status is a known prognostic and predictive factor with a more aggressive tumour biology but with an increased survival associated with anti-HER2 therapy [8] and the lack of this information complicates the interpretation of the OS results in current clinical practice. An additional limitation is the lack of information regarding treatment sequencing in metastatic setting since this information could not be captured through the Prescribed Drug Register. Future research should focus on developing the machine-learning classifier in order to be able to better distinguish between the different subgroups of MBC, most importantly in terms of HER2-, HR status, and subsequently molecular subtyping, and including other potential prognostic factors of interest to multivariable analyses. Further analysis of breast cancer-specific survival considering other causes of death as competing risk events is also an analysis of interest for future research using the stud cohort.

The method used in this study, and its inherent limitations may be compared to the comprehensive analysis of patients with MBC in France in the Unicancer ESME (Epidemiological Strategy and Medical Economics)-MBC national cohort [29, 30]. The ESME-MBC cohort is a population-based registry study collecting detailed information on patients with MBC treated at expert cancer centres. The ESME study purposely collects data to address research questions within MBC. The structured set-up and dedicated data acquisition allows for greater detail compared to our study methodology, e.g., molecular subtype, type of treatment and duration of response to treatment. However, the structured set-up approach is associated with higher costs. In comparison, this study used available health data, primarily collected for other purposes and represents a less costly methodology, although with a limited available level of detail compared to the ESME report. The use of the National population-based health register with close to 100% coverage avoids any patient selection that may bias outcomes which is a potential source of bias in ESME-cohort where only patients treated in specialised cancer centres are included.

## Conclusions

By use of machine learning applied to national registries, the median survival of patients with MBC diagnosed in Sweden during the period 2009 to 2016 was found to be approximately 30 months, with a 5-year survival rate of 30% and a 10-year survival rate of 14%. De novo metastatic may be associated with better survival, although the difference identified in this study was small, and caution should be used when interpreting the results due to the probable presence of uncontrolled confounding factors such as HER2 status. The results of this study may be used as a baseline to gauge the real-world effect of new treatments targeting MBC introduced after the study period, such as the CDK4/6- and PI3K-inhibitors targeting HR-positive MBC, and immunotherapy for triple-negative breast cancer. Finally, the observed improved survival has implications for healthcare resource use and decision-making—palliative breast cancer care needs to encompass survivorship, with a greater focus on the quality of life and co-morbidity management, as patients live longer.

## Supplementary information

Supplementary information

Reproducibility checklist

## Data Availability

The register data used is not publicly available as it contains sensitive information. To access the data, a request for extraction from the registers must be made to the National Board of Health and Welfare in Sweden. The National Board of Health and Welfare require an ethical approval to access the data.
