# Supplementary material for: Overall survival of patients with metastatic breast cancer in Sweden: a nationwide study
Source: Br J Cancer. 2022 May 21;127(4):720–5. doi: 10.1038/s41416-022-01845-z (PMC9381497; doi:10.1038/s41416-022-01845-z)
Supplement: Supplementary file 1 — Supplementary information [file 41416_2022_1845_MOESM1_ESM.docx]

**Supplementary information**

Overall survival of patients with metastatic breast cancer in Sweden: a nationwide study

Antonis Valachis, Peter Carlqvist, Yuanjun Ma, Máté Szilcz, Jonatan Freilich, Simona Vertuani, Barbro Holm, and Henrik Lindman

This supplementary information provides more detail regarding the development of the classifier used to identify patients with metastatic breast cancer in the national health registers. The development of the classifier has previously been published and more details may be found in Valachis *et al* (1).

**Development of the support vector machine classifier**

Swedish national data (National Patient Register, Prescribed Drug Register, Cancer Register and the Cause of Death Register) were linked with metastatic status, outcome, and biomarker data from a regional breast cancer register (Uppsala University Hospital) via unique personal identification number. These national registers contain complete data on all Swedish residents, and the personal identification number is used throughout the Swedish system, enabling direct data linkage. Patients included were defined as having a breast cancer diagnosis during 2009 to 2016.

The regional breast cancer register data, containing medical records of subjects with confirmed metastatic breast cancer together with subjects without metastasis during years 2009-2016, were divided into a training set (n=2,680) and a validation set (n=670). Predictor variables or features were derived from the national healthcare resource use registers, specifically the National Patient Register and the Prescribed Drug Register. Information on M1 status at diagnosis (*de novo* metastatic breast cancer) from the National Cancer Register was also included. Features used to train the two classifiers were selected by a clinical expert in breast cancer (HL) and a detailed analysis of available features in the registers, features showing high variability between subjects with metastatic breast cancer and subjects without metastases were prioritised for inclusion in the classifiers. The full list of features is presented in the Supplemental material accompanying the publication of the classifiers (1).

The training set was used to develop two classifiers, a Classification and Regression Tree (CART) and a support vector machine (SVM). A CART decision tree repeatedly splits the data into two branches based on one variable at the time. SVM is a method that separates data using a hyperplane, i.e. it attempts to find the optimal separation of data points in a multidimensional space. The classifiers were optimised using the training set and their performance evaluated using the validation set.

The SVM was identified as giving the least false positives and was chosen for application to the national breast cancer population. A schematic of procedure is presented in Figure S1.

Figure 1. Schematic of the procedure to identify patients with metastatic breast cancer in Sweden


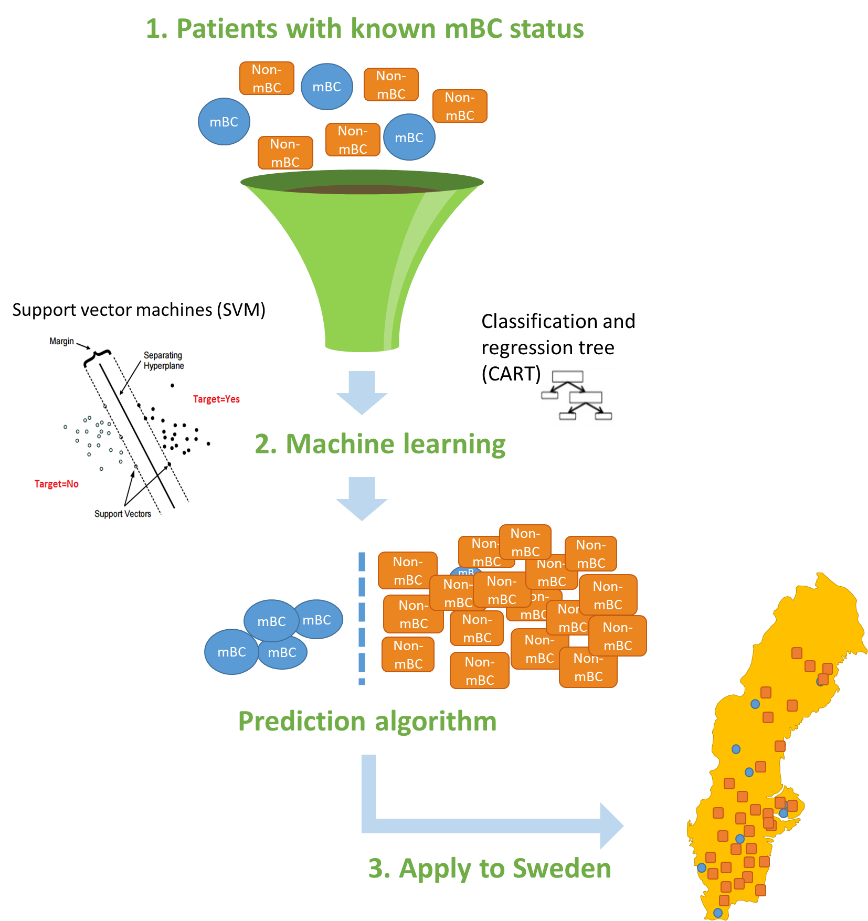


**References**

1. Valachis A, Carlqvist P, Szilcz M, Freilich J, Vertuani S, Holm B, et al. Use of classifiers to optimise the identification and characterisation of metastatic breast cancer in a nationwide administrative registry. Acta Oncol 2021(1651-226X (Electronic)).
